# Optimal circulating high‐density lipid concentration is associated with lower midlife white matter hyperintensity volume: The Bogalusa Heart Study

**DOI:** 10.1002/alz70860_100496

**Published:** 2025-12-23

**Authors:** Christian P Corsten, Franklin M Epps, Ariana M Duran, Lydia A Bazzano, Owen T. Carmichael

**Affiliations:** ^1^ Pennington Biomedical Research Center, Baton Rouge, LA, USA; ^2^ Celia Scott Weatherhead Tulane University School of Public Health and Tropical Medicine, New Orleans, LA, USA

## Abstract

**Background:**

Reports of the relationship between circulating serum cholesterol components (high‐density lipid or HDL, low‐density lipid or LDL, and triglycerides) and brain health in midlife and late life have been mixed. We aimed to assess the association between cholesterol components in the fourth and fifth decades of life, and MRI‐based white matter hyperintensity (WMH) volume in the sixth decade of life, in a community‐based cohort.

**Method:**

The Bogalusa Heart Study collected longitudinal blood samples from 1973 to 2024, and brain MRI from a subset of participants 2018 to 2024. Serum‐based HDL, LDL, and triglycerides were averaged within participants at visits that started in their early 30s (mean age at first visit: 31.0 ± 4.1) and ended in their early forties (mean age at last visit: 42.9 ± 4.2). Participants were categorized according to HDL, LDL, and triglyceride levels being within or outside of the optimal range (i.e., HDL ≥ 60 mg/dL, LDL < 100 mg/dL triglycerides < 150mg). MRI‐based WMH volume, which was measured when participants were in their mid‐fifties (mean age at MRI: 54.8 ± 4.3), was compared between these cholesterol component categories in ANOVA models controlling for sex, race, and age.

**Result:**

MRI recipients (*n* = 170) were predominantly female (66%) and Caucasian (83%). The optimal HDL group had lower WMH volume than the sub‐optimal HDL group (mean difference: ‐0.249 cm^3^, CI: [‐0.4734, ‐0.0242], *p* = 0.0301). No significant differences in WMH volume were observed between LDL categories or triglyceride categories.

**Conclusion:**

Optimal mean serum HDL concentrations in the fourth and fifth decades of life may be associated with reduced WMH burden in midlife. Contributions of separate cholesterol components to midlife brain health are worthy of further study.

